# Porous Hexagonal Boron Nitride as Solid-Phase Microextraction Coating Material for Extraction and Preconcentration of Polycyclic Aromatic Hydrocarbons from Soil Sample

**DOI:** 10.3390/nano12111860

**Published:** 2022-05-29

**Authors:** Dan Li, Mengyuan Li, Shiping Zhu, Yanmei Gao, Mengyao Mu, Ning Zhang, Youmei Wang, Minghua Lu

**Affiliations:** Henan International Joint Laboratory of Medicinal Plants Utilization, College of Chemistry and Chemical Engineering, Henan University, Kaifeng 475004, China; nlidan@yeah.net (D.L.); limyzz@yeah.net (M.L.); zhushiping2022@yeah.net (S.Z.); gyanmei@yeah.net (Y.G.); mumengyao0923@yeah.net (M.M.); ymwanghenu@yeah.net (Y.W.)

**Keywords:** sample pretreatment, solid-phase microextraction (SPME), hexagonal boron nitride (h-BN), nanomaterials, polycyclic aromatic hydrocarbons (PAHs), soil

## Abstract

Sample pretreatment plays important role in the analysis and detection of trace pollutants in complex matrices, such as environmental and biological samples. The adsorption materials of sample pretreatment receive considerable attention, which has a significant effect on the sensitivity and selectivity of the analytical method. In this work, the porous hexagonal boron nitride (h-BN) was utilized as a coating material of solid-phase microextraction (SPME) to extract and preconcentrate polycyclic aromatic hydrocarbons (PAHs) prior to separation and detection with GC-FID. Attributed to the multiple interactions including hydrophobicity, hydrogen bonding and strong π–π interaction, the h-BN coating showed excellent extraction performance for PAHs. Under the optimal conditions, the method showed the linear relationship in the range of 0.1–50 ng mL^−1^ for acenaphthene, 0.05–50 ng mL^−1^ for pyrene, and 0.02–50 ng mL^−1^ for fluorene, phenanthrene and anthracene with a correlation coefficient (*R*^2^) not lower than 0.9910. The enrichment factors were achieved between 1526 and 4398 for PAHs with h-BN as SPME fiber coating. The detection limits were obtained in the range of 0.004–0.033 ng mL^−1^, which corresponds to 0.08–0.66 ng g^−1^ for soil. The method was successfully applied to analysis of real soil samples. The recoveries were determined between 78.0 and 120.0% for two soil samples. The results showed that h-BN material provided a promising alternative in sample pretreatment and analysis.

## 1. Introduction

Polycyclic aromatic hydrocarbons (PAHs), as a kind of persistent organic pollutants, contain two or more benzene rings in their molecules. They are mainly produced by the incomplete combustions of coal, fossil oil, gasoline, diesel and tobacco, as well as the frying and barbecuing of food [1,2,3]. PAHs can continuously migrate in the environment and enter the human body through various routes, such as breathing and skin contact, as well as food and drinking water ingestion [4]. Due to PAHs possessing potential mutagenic, carcinogenic and teratogenic effects, long-term exposure to PAHs may cause serious health risks to humans and other organisms [5]. In view of the widespread presence in the environment and the prominent adverse health risks to humans, it is very necessary to establish an efficient, sensitive and rapid method to detect PAHs. However, due to their trace or ultra-trace property and coexistence with various interferences in environmental or biological samples, the analysis and detection of PAHs received great challenges [6,7,8]. Sample pretreatment is considered as an effective way to improve the sensitivity of the method for analysis and detection of trace PAHs in complex samples, with the elimination of interferences and precontraction of targets.

As one of the most important procedures for the analysis of trace targets in complex matrices, sample pretreatment not only influences the sensitivity, selectivity, accuracy and repeatability of the analytical technique, but also the total analysis time of the method. Over past few decades, various sample pretreatment techniques, such as Soxhlet extraction, supercritical fluid extraction, ultrasonication-assisted extraction, liquid-phase microextraction and solid phase extraction, were developed for clean-up and enrichment of environmental pollutants [9,10,11,12,13]. Owing to easy operation, excellent adsorption performance and plenty of materials able to be used as sorbents, solid phase-based extraction techniques, including conventional solid phase extraction [14], dispersive solid phase extraction [15,16,17], magnetic solid phase extraction [18,19,20,21], stir bar sorptive dispersive microextraction [22] and solid-phase microextraction (SPME) [23,24,25,26] received considerable attention in environmental and biological analysis. 

Due to the integration of sampling, extraction, concentration and injection into one step, as well as not requiring or only consume a small amount of organic solvents, SPME usually is considered an environmentally friendly sample pretreatment technology [27,28,29,30,31,32]. However, commercial fiber coatings, including polydimethylsiloxane, polyacrylate, carboxen/divinylbenzene and polyacrylonitrile, can be selected for SPME [33]. Due to tremendous difference in chemical and physical properties of target analytes, the commercial fiber coatings have some difficulty in meeting the requirement for all compounds. Therefore, various new coating materials including graphene composites [34], graphitic carbon nitride [35], metal-organic frameworks [36] and covalent organic frameworks [37] have been exploited as SPME coating materials. However, the preparation processes of these materials mostly require organic reagents with tedious preparation steps. Therefore, the environmentally friendly preparation of coating materials with excellent enrichment properties is of great significance for the development of SPME technology.

Porous hexagonal boron nitride (h-BN) nanorods as a new type material possess a graphene-like crystal structure [38]. Benefiting from the good thermal and chemical stability, high specific surface area, large pore volume and abundant active sites [39], h-BN nanorods show excellent performance in catalyst support [40], wastewater treatment [41], energy storage and gas adsorption [42]. Importantly, the preparation process of h-BN nanorods is carried out in aqueous solution without any organic solvent, avoiding the new pollution problems accompanying the treatment process. In view of their inherent properties, h-BN nanorods are expected to be an ideal candidate as a fiber coating material of SPME.

In this work, the porous h-BN was prepared and used as coating material of SPME for the extraction and preconcentration of PAHs. Five PAHs, including acenaphthene (ACE), fluorene (FLU), phenanthrene (PHE), anthracene (ANC) and pyrene (PYR), were selected to evaluate the performance of porous h-BN as an SPME fiber coating material. The parameters affecting extraction efficiency were investigated and the mechanism for the SPME of PAHs was also discussed. Finally, the h-BN as fiber coating material combined with direct immerse SPME was applied to extract and enrich trace PAHs from soil sample prior to their GC-FID analysis. 

## 2. Materials and Methods

### 2.1. Reagents and Standards

The PAHs, including ACE, FLU, PHE, ANC and PYR as standard compounds, were purchased from Aladdin (Shanghai, China). Tetraethyl orthosilicate (TEOS, C_8_H_20_O_4_Si), boric acid (H_3_BO_3_) and melamine (C_3_H_6_N_6_) were obtained from Aladdin (Shanghai, China). Acetone with HPLC grade was supplied by Merck (Darmstadt, Germany). Dehydrated alcohol (EtOH) was purchased from Anhui Ante Food. Co., Ltd. (Suzhou, China). Hydrochloric acid (36%) and nitric acid (98%) were received from Kermel chemical reagent Co., Ltd. (Tianjin, China). The stainless-steel fibers were acquired from Hengwang Metal Material Factory (Guangzhou, China). Ultrapure water purified with Milli-Q purification system (Millipore, Bedford, MA, USA) was used throughout the experiments. All reagents and standards were used without further purification.

### 2.2. Instruments 

All chromatographic analysis were carried out on an Agilent 7890B GC (Agilent, Santa Clara, CA, USA) equipped with a flame ionization detector (FID). The analytes were separated with a HP-5 capillary column (30 m, 0.32 mm, 0.25 μm). The programmed temperature of gas chromatography was as follows. The initial temperature was set at 60 °C and held for 1 min, increased to 190 °C at the rate of 50 °C min^−1^ and maintained for 1 min, then increased by 6 °C min^−1^ to 220 °C and kept for 0.5 min, and finally increased to 300 °C at the rate of 80 °C min^−1^, where it was maintained for 2 min. The temperature of the injector and detector were both set at 300 °C. High-purity nitrogen (99.99%) was used as a carrier gas, with the splitless mode at a constant flow rate of 1 mL min^−1^. 

The morphology of h-BN nanorods was characterized by scanning electron microscope (SEM, JSM-7610 F, JEOL, Tokyo, Japan) at 5 kV and transmission electron microscopy (TEM, JEM 2100, JEOL). Fourier transform infrared spectroscopy was carried out by using a vertex 70 spectrometer (Bruker, Saarbrücken, Germany). A Bruker D8 Advance diffractometer (Cu kα) (Bruker, Karlsruhe, Germany) was used to record the powder X-ray diffraction (PXRD) patterns of the materials. The surface area and porosity analyzer of h-BN nanorods were calculated by nitrogen adsorption analytical isotherm, according to Brunauer Emmett Teller (BET) method (ASAP 2020, micromeritics, Norcross, GA, USA). Thermogravimetric analysis (TGA, QMS 403 D Aëolos^®^, NETZSCH, Selb, Germany) was used to evaluate the thermal stability of h-BN nanorods from room temperature to 800 °C at a heating rate of 10 °C min^−1^ under a nitrogen atmosphere. The magnetic stirrer (IKA, Staufen, Germany) was used for stirring and SB-120 DT ultrasonicator (Ningbo Scientz Biotechnology, Ningbo, China) was used for strip bulk materials. The analytes were accurately weighed using a SQP Sartorius analytical balance (Beijing, China). 

### 2.3. Synthesis of h-BN Nanorods

The h-BN nanorods were synthesized according to the previously reported method with minor modifications [43]. Firstly, C_3_N_6_H_6_·2H_3_BO_3_ precursor was synthesized. The specific steps were as follows: 0.06 mol of boric acid (H_3_BO_3_) and 0.02 mol of melamine (C_3_N_6_H_6_) were added to a beaker containing 200 mL of ultrapure water. The mixture was stirred in a water bath of 85 °C for 2 h, lowered to 70 °C for 12 h and finally stirred at 55 °C for 8 h. After cooling down to room temperature, the precipitation was washed with ultrapure water and dried overnight in a vacuum oven at 60 °C. Then, to synthesize h-BN nanorods, the obtained precursor was sintered in a 1000 °C tubular furnace for 3 h at a heating rate of 10 °C min^−1^ under a flowing nitrogen atmosphere (80 mL min^−1^). 

### 2.4. Preparation of Sol-SiO_2_

In order to obtain h-BN-coated fiber, sol-SiO_2_ was synthesized according to our previous work [44]. 2 mL of tetraethyl silicate (TEOS) and 3.4 mL of ethanol (EtOH) were firstly mixed, 0.6 mL of ultrapure water and 0.15 mL of HCl (pH = 3–4) were then added dropwise under vigorous stirring. Finally, the solution was sonicated for 30 min.

### 2.5. Preparation of h-BN Coated Fiber and Stability Test 

**Figure 1:**
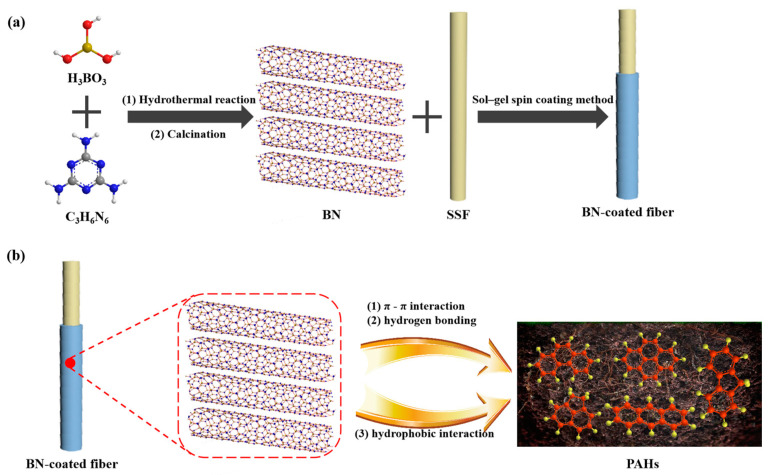
Schematic illustration of the fabrication of h-BN-coated SPME fiber (**a**) and its extraction process (**b**).

The preparation process of h-BN-coated fiber was shown in Figure 1. In brief, one end of the fiber (about 2 cm) was immersed in a mixture of hydrochloric acid and nitric acid (1:3; *v*/*v*) for 3 min to obtain a rough surface, washed with water and dried. Secondly, the etched part of fiber was immersed in sol-SiO_2_ solution for 1 min, rolled three times in fine h-BN powder and then placed in an oven. The second process was repeated 12–15 times to ensure a uniform coating. Finally, the fiber was loaded into an extraction apparatus consisting of a 5 µL microsyringe and aged at 300 °C for 1 h at the GC injector to remove surface impurities. 

In addition, the stability of fiber coating was conducted by immersing the h-BN coating in water for different time interval (12 to 48 h) or for 12 h at different organic solvents (ethanol, methanol, acetonitrile and n-hexane).

### 2.6. Standard Solution and Real Sample Preparation

The PAHs, including ACE, FLU, PHE, ANC and PYR, were ultrasonically dissolved in acetone and configured as the single stock solution with a mass concentration of 1 mg mL^−1^. The mixed reserve standard solution of 2 μg mL^−1^ was prepared by diluting each standard solution (1 mg mL^−1^) step by step with acetone. The prepared solutions were stored in a refrigerator at 4 °C.

The soil samples were collected from Zhengkai Avenue (Kaifeng, China) and campus of Henan University (Kaifeng, China), respectively. The soil samples were dried at room temperature for seven days and ground to pass through 200-mesh sieve. Then, 30 g soil was added to 40 mL acetone with vortex for 5 min and sonication for 40 min. After extraction, the suspension was centrifuged at 8000 rpm for 10 min. Extracted again, the supernatant layer of twice were collected. All the supernatants were mixed and purged to dryness in an oven at 60 °C. The dry residue was redissolved with 3 mL acetone as a soil extract. Then, 50 μL of above soil extract was diluted to 10 mL with salt solution (NaCl, 20%, *w*/*v*) for real sample analysis and recovery experiment.

### 2.7. SPME Procedures

The SPME process was carried out on a direct immersion model. Due to soil sample with absolute PAHs free is very difficult to obtain, the ultrapure water replaced soil extract was used to optimize SPME extract conditions and prepare calibration curve. In the processes of SPME, a concentration of 20 ng mL^−1^ by spiking standard solution to 10 mL salt solution (NaCl, 20%, *w*/*v*) was used to optimization. The salt solution, including spiked standard solution or soil extract, was put into a 20 mL glass vial, and immediately capped with a PTFE-coated septum. During the extraction process, the fiber coating was immersed into the stirred solution for a certain period of time (30–80 min). The extraction temperature between 20 and 40 °C was controlled with a thermostatic water bath. Magnetic stirring in the range of 400 to 900 rpm was carried out to agitate the solution with a Teflon-coated stir bar. After extraction, the fiber was removed from the glass bottle and immediately inserted into the GC inlet for thermal desorption at 250–300 °C for 0.5–3 min. Before two consecutive extractions, the fiber was conditioned at 300 °C for 5 min. 

### 2.8. Protocols for Method Validation 

Calibration curves were experimentally determined by external standard curve method with spiking PAHs into salt solution (NaCl, 20%, *w*/*v*), which was prepared by ultrapure water under the optimized condition. The calibration plots were achieved at different concentration levels (50, 30, 10, 2.5, 0.5, 0.1, 0.05 and 0.02 ng mL^−1^) by the developed method. For each concentration point, three replicate extractions were performed. The calibration curves were obtained by plotting peak areas of the analytes against their corresponding concentrations. The limits of detection (LODs) and limits of quantification (LOQs) were calculated based on signal-to-noise ratios of 3 and 10, respectively. The intra-day precision was evaluated by three repeated experiments in one day with the same fiber. The inter-day precision was performed in three consecutive days for three analyses with the same fiber. The fiber-to-fiber precision was investigated on three different fibers which obtained with different batches. All precision analyses were investigated by analyzing standard solution with a concentration of 20 ng mL^−1^. 

The soil extract with a volume of 50 μL which corresponding 0.5 g soil was diluted to 10 mL with salt solution (NaCl, 20%, *w*/*v*) for real sample analysis. The samples that were used for the recovery test were prepared by mixing 50 μL soil extract with different volumes (2.5, 25 and 100 μL) of standard solution (2 μg mL^−1^) and diluted to 10 mL by salt solution (NaCl, 20%, *w*/*v*), corresponding to 10, 100 and 400 ng g^−1^ in the soil sample, respectively. The recovery was calculated based on the following equation

Recovery = (*M*_t_ − *M*_b_)/*M*_s_ × 100%

where *M*_t_ and *M*_b_ refer to the mass of PAHs that determined by developed method in spiked and blank soil samples, respectively. The *M*_s_ is the mass of PAHs that spiked to sample. The blank contents were having into account to calculate the recoveries.

## 3. Results and Discussion

### 3.1. Characterization of h-BN

**Figure 2:**
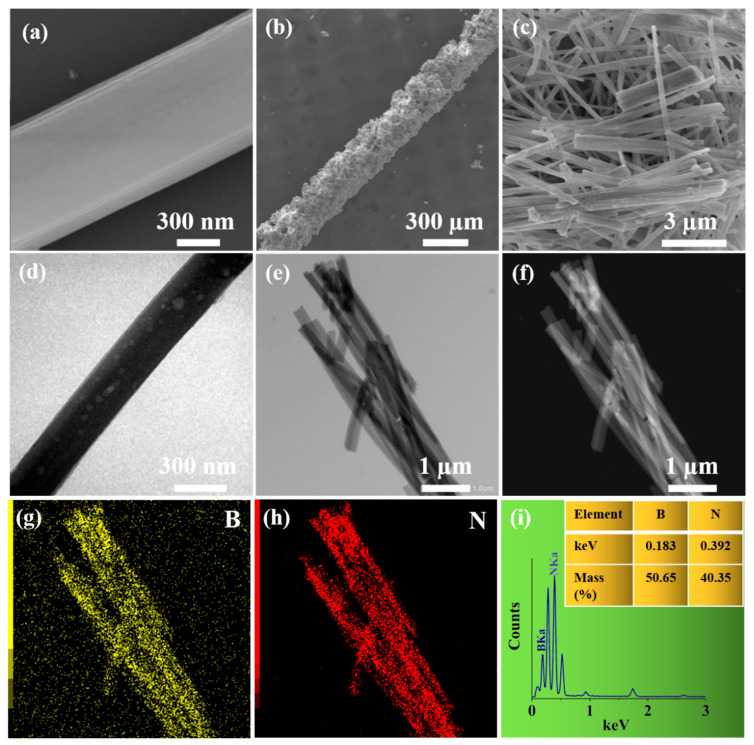
SEM of bare fiber (**a**); h-BN-coated fiber (**b**); h-BN coating (**c**); TEM of h-BN (**d**,**e**); elemental mapping of the h-BN (**f**–**h**); and the corresponding EDS spectra of h-BN (**i**).

The morphology and structure of h-BN-coated fiber were characterized by SEM and TEM. As shown in Figure 2a, the bare fiber displayed a smooth surface with an approximate diameter of 130.36 µm. After the physical coating process, a homogenous, porous and dense h-BN coating was observed on the surface of the fiber (Figure 2b). Calculated from Figure 2a,b, the h-BN layer possessed an approximate diameter of 91.90 µm. As shown in Figure 2c, the h-BN coating owed a fibrous morphology and porous structure, which would facilitate the mass transfer and the extraction process in the process of SPME [45]. Detailed TEM analysis further revealed the porous nature of the nanorods. In Figure 2d,e, the TEM image proved that h-BN showed the fibrous structure and the diameter of a single h-BN fiber was about 251 nm. The results of their structure and morphology are very similar with that of other report [46]. High-angle annular dark-field TEM images (Figure 2f) and elemental mapping (Figure 2g,h) further demonstrated the uniform distribution of B and N elements in h-BN coating. The corresponding EDS spectrum (Figure 2i) showed that the mass percentages of B and N were 50.65% and 40.35%, respectively. 

**Figure 3:**
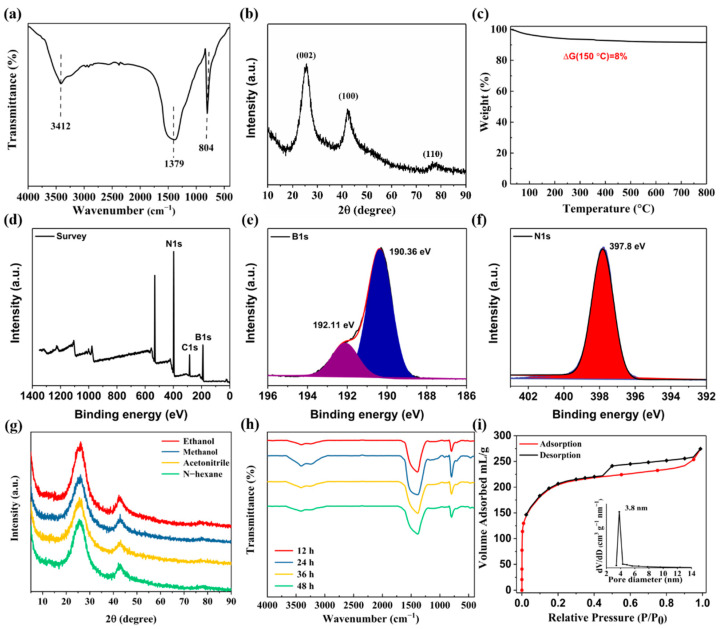
The XRD pattern (**a**), FT-IR spectra (**b**), TGA curve (**c**) of h-BN, the XPS spectrum of h-BN (**d**), the B1s (**e**) and N1s (**f**) spectra of h-BN, the XRD patterns of h-BN after soaking in the different organic solvents for 12 h (**g**); the FT-IR patterns of h-BN after soaking for 12 h, 24 h, 36 h and 48 h in the water (**h**); and the nitrogen-adsorption isotherms and pore diameter distribution of h-BN (**i**).

The FT-IR spectra of h-BN nanorods is presented in Figure 3a. The absorption band at 3412 cm^−1^ is attributed to the stretching vibration of –OH, which might come from water molecules adsorbed on the h-BN surface. The absorption peak at 1379 cm^−1^ is related to B–N stretching vibration, and the absorption peak at 804 cm^−1^ is associated with B–N–B bending vibration. The structure of h-BN nanorods is characterized by XRD (Figure 3b). The three characteristic peaks are labeled at 3.41 Å (002), 2.17 Å (100), 1.25 Å (110), which proved that the material owed a typical h-BN structure. The results of XRD were consistent with other report for characterization of h-BN [47,48]. In order to study the thermal stability of h-BN nanorods, thermogravimetric analysis was carried out. It can be observed from Figure 3c, the weight loss is about 8% at 150 °C, and then kept in equilibrium between 150 and 800 °C, the weight loss (8%) might be caused by the water loss on the material surface. The high thermal stability meant that the h-BN material could be stable in the SPME thermal desorption process. The XPS analysis was used to determine chemical elements and bonding forms of materials. The full XPS spectrum (Figure 3d) exhibits strong signals of B and N elements of the h-BN. In addition, the detected carbon information in Figure 3d can be attributed to surface contamination, which is commonly observed by the sensitive XPS method [49]. High-resolution XPS spectra of B and N components are shown in Figure 3e,f. The B 1s spectrum of h-BN was fitted to two peaks at 190.36 and 192.11 eV, attributing to the original h-BN compound (190.36 eV) and the intermediate species (192.11 eV), respectively (Figure 3e) [50]. In the N 1s core-level spectra of h-BN (Figure 3f), the N 1s spectrum was fitted to one peak ascribed to the N−B bonds [50]. From the XPS spectra, the atomic percentages of B and N elements were 51.03% and 35.57%, which was consistent with the data obtained from the EDS spectrum. Figure 3g,h show that the characteristic peaks of h-BN coating under various organic solvents for 12 h or at different immerse time in water. The results demonstrated that the material presented good solvents and water stability. The excellent water and solvent stability of h-BN may be related with its layered structure similar to graphite, which played a “protective screen” role with strong stability and hydrophobicity. The satisfied water and solvent stability of as-prepared h-BN coating was of great benefit for the improvement of fiber lifetime in DI-SPME. In order to study the specific surface area and pore size distribution of h-BN nanorods, N_2_ adsorption-desorption isotherms were measured. From Figure 3i, it could be seen that h-BN nanorods exhibited type-IV isotherms adsorption equilibrium with an IV hysteresis loop, indicating the presence of a mesoporous structure. Furthermore, the nitrogen adsorption isotherm and pore diameter distribution of h-BN coating illustrated a high surface area of 680 m^2^ g^−1^ and a uniform mesopore distribution (3.8 nm), which could offer more potential interaction sites and spatial match effects for the extraction of target analytes.

### 3.2. Optimization of SPME Experimental Conditions

To achieve the optimized adsorption and desorption conditions for extraction of PAHs with h-BN as fiber coating, the experimental parameters, including extraction temperature, extraction time, desorption temperature, desorption time, agitation speed and ionic strength, were optimized. 

#### 3.2.1. Extraction Temperature

**Figure 4:**
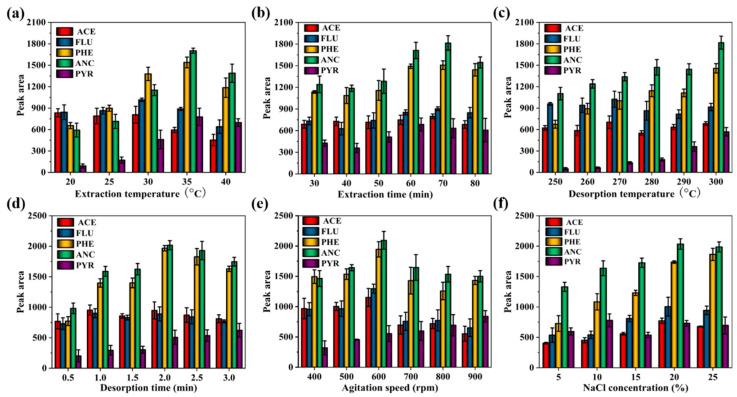
The influence of experimental conditions on extraction efficiency for the PAHs obtained on the h-BN-coated fiber. Extraction temperature (**a**); extraction time (**b**); agitation speed (**c**); desorption temperature (**d**); desorption time (**e**); and NaCl concentration (**f**).

In the processes of SPME, the extraction temperature has a significant effect on the extraction efficiency. The extraction temperature can affect the partition coefficient between SPME fiber coating material and targets. Generally, increasing the temperature can enhance mass transfer process of analytes from the aqueous phase to fiber coating material, which resulting in faster extraction [51]. However, since most adsorption is generally an exothermic process, too high a temperature is not a favorite to adsorption of analytes on fiber coating. The extraction temperature was investigated from 20 to 40 °C with keeping extraction time at 70 min, desorption temperature at 300 °C, desorption time at 2 min, agitation speed at 600 rpm and salt concentration at 20% (NaCl, *w*/*v*). Figure 4a demonstrates that the peak areas reached maximum value at 35 °C. Therefore, 35 °C was selected as the best temperature for subsequent experiments.

#### 3.2.2. Extraction Time

Extraction time is an important factor to achieve the distribution balance of targets between coating and sample solution [52]. Generally, the adsorption amount will increase with extending adsorption time before equilibration. To investigate the extraction time effect on adsorption amount, the experimental parameters, including extraction temperature, desorption temperature, desorption time, agitation speed and salt concentration, were set as 35 °C, 300 °C, 2 min, 600 rpm and 20% (NaCl, *w*/*v*). As shown in Figure 4b, the adsorption amount gradually increased from 30 to 70 min, and has a slight decrease when extraction time is further exceeded to 80 min. Therefore, 70 min was selected as the optimal adsorption time.

#### 3.2.3. Desorption Temperature

Desorption temperature is another important parameter in the process of SPME. The targets cannot be completely desorbed from fiber coating material at a lower desorption temperature, but too high a temperature would destroy the stability and shorten the life of fiber coating [26]. The desorption temperature was studied in the range from 250 to 300 °C, with extraction temperature, extraction time, desorption time, agitation speed and salt concentration of 35 °C, 70 min, 2 min, 600 rpm and 20% (NaCl, *w*/*v*), respectively. From Figure 4c, the peak areas of analytes increased with the enhancing desorption temperature. Generally, excessive temperature will shorten the life of the extracted fibers. Therefore, in order to protect the fibers, 300 °C was selected as the optimal desorption temperature.

#### 3.2.4. Desorption Time

The desorption time is related to desorption temperature and thickness of fiber coating material. To completely desorb analytes from SPME fiber coating material and reduce the residuals of targets on fiber coating, sufficient desorption time is usually required. However, a long desorption time would affect the peaks shape and lifetime of fiber coating [32]. Keeping other SPME parameters constant (such as extraction temperature: 35 °C, extraction time: 70 min, desorption temperature: 300 °C, agitation speed: 600 rpm and salt concentration: 20% (NaCl, *w*/*v*)), the desorption time was studied from 0.5 to 3 min, the and results were displayed in Figure 4d. It could be seen that the peak areas of the analytes reached the maximum at 2 min. Thus, 2 min was selected as the desorption time for further experiments.

#### 3.2.5. Agitation Speed

Agitation speed is another factor affecting the extraction efficiency. The transfer rate of targets from solution to fiber coating can be accelerated with increasing agitation speed [3]. However, the vortex produced by high agitation speed is not favorite for effective contact of analytes with fiber coating. Moreover, the equilibration between targets and fiber coating will be destroyed with high agitation speed. To evaluate the agitation speed between 300–800 rpm, the extraction temperature, extraction time, desorption temperature, desorption time and salt concentration were set as 35 °C, 70 min, 300 °C, 2 min and 20% (NaCl, *w*/*v*), respectively. The result was presented in Figure 4e. The peak areas of PAHs reached the maximum with the agitation speed up to 600 rpm, and then decreased with the further increase in the agitation speed. Therefore, an agitation speed of 600 rpm was chosen for subsequent studies.

#### 3.2.6. Ionic Strength

In the process of SPME, high ionic strength in solution is usually beneficial to adsorption with decreasing solubility of targets in the solution. However, the ion strength also effects the equilibrium constant between fiber coating material and target analytes and increasing the viscosity of solution, which is not to benefit of extraction. Thus, a suitable ionic strength is usually required to investigation. To evaluate the ionic strength effect on adsorption, NaCl with the content ranging 5% to 25% in solution was studied under extraction temperature at 35 °C, extraction time at 70 min, desorption temperature at 300 °C, desorption time at 2 min and agitation speed at 600 rpm. It could be seen from Figure 4f that the best adsorption performance was obtained when the salt concentration was set at 20% (*w*/*v*). From the above experiment, the salt concentration was chosen at 20% in the follow-up experiments.

In brief, the optimum experiment conditions for PAHs determined by SPME-GC-FID were set as follows: extraction temperature: 35 °C; extraction time: 70 min; desorption temperature: 300 °C; desorption time: 2 min; agitation speed: 600 rpm; NaCl: 20% (*w*/*v*). 

### 3.3. Method Evaluation

To evaluate the performance of the developed DI-SPME-GC-FID method by using h-BN as fiber coating material for analysis of PAHs, the linear range, correlation coefficients (*R*^2^), limits of detection (LOD), limits of quantification (LOQ), enrichment factor (EF) and relative standard deviations (RSDs) were determined under the optimized conditions. The results were presented in Table 1. The method showed the linear relationship in the range of 0.1–50 ng mL^−1^ for ACE, 0.05–50 ng mL^−1^ for PYR and 0.02–50 ng mL^−1^ for FLU, PHE and ANC with a correlation coefficient (*R*^2^) not lower than 0.9910. The limits of detection (LODs) were obtained in the range of 0.004–0.033 ng mL^−1^, which correspond to 0.08–0.66 ng g^−1^ for soil. The limits of quantification (LOQs) were in the range of 0.02–0.10 ng mL^−1^, which correspond to 0.40–2.00 ng g^−1^ for soil. The RSDs of intra-day (*n* = 3) and inter-day (*n* = 3) were obtained in the ranges of 3.4–6.4% and 4.6–11.8%, respectively. The fiber-to-fiber reproducibility (*n* = 3) were 5.6–8.8%. In addition, the extraction performance of SPME fiber coating material was not obviously decrease during 80 adsorption and desorption cycles. The good durability of fiber coating may benefit from the excellent thermal stability of h-BN material. 

### 3.4. Enrichment Factor and Extraction Mechanism 

The enrichment factor (EF) is usually applied to investigate extraction ability of SPME fiber coating. The calculation formula of the enrichment factor is determined as *EF* = *C*_F_/*C*_S_, where *C*_F_ is the concentration of the measured analytes after extraction and enrichment and *C*_S_ is the concentration of the measured compounds in the original solution. EFs were calculated between 1526 and 4398 according to the formula, and the results were listed in Table 2. 

The h-BN usually consists of a few layers through sp^2^ hybridization. The structure of h-BN is similar to that of graphene or C_3_N_4_. Due to B–N bond, the h-BN is a polar material with uneven charge distribution. The target analytes of PAHs favored to be adsorbed on the surface of h-BN through the top^N^ sites by co-planar configuration with the aromatic rings nearly parallel to the h-BN surface [53,54]. The logK_ow_ is usually used to describe the hydrophobicity of analytes and the logK_ow_ of the target PAHs are presented in Table 2. The EFs was improved with increasing of logK_ow_ except PYR, which indicates that the hydrophobic interaction between h-BN and PAHs has significant effect on adsorption. The reduced EFs of PYR may be related with physical-chemical property and structure. The similar results were also reported by Doong and Chang, that the high molecular weight PAHs were less effectively extracted by polar coating material due to low affinity to high-ring PAHs [55]. Moreover, a large number of N atoms in h-BN network can produce hydrogen bond interaction with PAHs. Therefore, the adsorption may be controlled by synergistic effects of π–π interaction, hydrophobic interaction and hydrogen bond between h-BN and targets.

### 3.5. Analysis of Real Soil Samples

**Figure 5:**
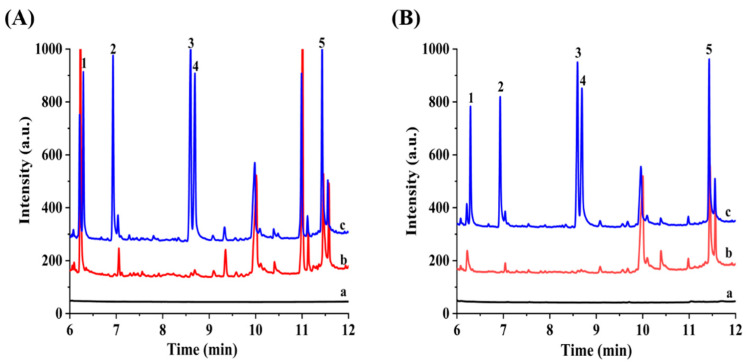
Typical chromatograms obtained for the analysis of campus soil (**A**) and Zhengkai Avenue soil (**B**) with different methods. Direct inject extract of blank soil with a volume of 1 μL (a), the extract of blank soil (b) and spiked soil (400 ng g^−1^) (c) pretreated with developed SPME-GC-FID using h-BN as fiber coating. Peaks identification: (1) ACE, (2) FLU, (3) PHE, (4) ANC and (5) PYR.

In order to verify the applicability of the developed method, two real soil samples were determined under the optimized conditions. For easy comparison, the standard solution of PAHs (20 ng mL^−1^, 1.0 µL) was firstly analyzed by the direct injection model. As shown in Figure 5 (chromatogram a), no peak was observed in the chromatogram. After pretreatment with SPME by using h-BN as fiber coating, four target analytes (FLU, PHE, ANC and PYR) were detected in the two real samples. The contents of FLU, PHE, ANC and PYR ranged from 6.4–255.6 ng g^−1^ for campus soil and 5.6–251.0 ng g^−1^ for Zhengkai Avenue soil, respectively. 

To evaluate the matrix effects, the soil samples were spiked with standard solution of PAHs at three different concentration levels (10, 100 and 400 ng g^−1^). The typical chromatograms of soil samples with spiking 400 ng g^−1^ were presented in Figure 5 (chromatogram c). There no obvious interference was observed from chromatogram c in Figure 5, which can be attributed that the adsorption of SPME fiber coating material toward PAHs has some selectivity. Moreover, the relative recoveries were calculated to be from 78.0% to 120% for two soil samples with RSDs in the range of 0.1–9.8%. As shown in Table 3, the detected contents and the obtained relative recoveries demonstrated that both accuracy and precision are acceptable for soil samples.

### 3.6. Comparison with Other Fiber Coating Materials 

The developed method by using h-BN as SPME fiber coating material was compared with other reported techniques for the analysis of PAHs in soil samples (Table 4). It can be seen that the developed GC-FID method has equal or better LODs with that of GC-MS technique [56,57]. Since high temperature is usually required for desorption in the process of SPME, the thermal stability of material is an important parameter to evaluate the performance of fiber coating. The h-BN as SPME fiber coating material showed better thermal stability than that of carbon nanospheres [58], BN@rGO [59] and MWCNTs/MnO_2_/PEDOT [60]. A wider linearity range were obtained with developed method for analysis of PAHs in soil samples compared with other reported techniques. The excellent extraction performance was mainly attributed to the larger specific surface area of h-BN could provide more accessible active sites, accompanied by improvement the hydrophobic interaction, π–π interaction and hydrogen bonding interaction between h-BN coating and PAHs.

## 4. Conclusions

In this work, porous h-BN material was prepared and used as the coating material of SPME. Due to its high specific surface area, good thermal and humidity stability, h-BN as SPME fiber coating exhibited excellent adsorption property and strong preconcentration ability to PAHs. Benefiting from the strong electrostatic interaction, hydrogen bonding and π–π stacking between h-BN coating and the PAHs, the established method exhibited a wider linear range, lower LODs and higher sensitivity. The developed SPME-GC-FID method was successfully applied to determination of PAHs in soil samples. The fiber could be used up to 80 times without significant decrease in adsorption performance. A sensitive, simple, reliable, low-cost and environmentally friendly method for the detection of trace PAHs in soil was established by combining the h-BN-coated fiber and GC-FID. The ability to recognize PAHs from complicated soil samples offered the proposed SPME-GC-FID method promising potential for the residual monitoring and assessment of PAHs.

## Figures and Tables

**Table 1:** Related analytical parameters of the developed SPME-GC-FID by using h-BN as fiber coating material for determination of PAHs.

Analyte	Linear Range (ng mL^−1^)	*R* ^2^	LOQs (ng mL^−1^/ng g^−1^) ^a^	LODs (ng mL^−1^/ng g^−1^)	RSD% (*n* = 3)		
					Intraday	Interday	Fiber-to-Fiber
ACE	0.1–50	0.9911	0.10/2.00	0.033/0.66	5.94	10.40	6.14
FLU	0.02–50	0.9910	0.02/0.40	0.004/0.08	3.36	10.07	7.46
PHE	0.02–50	0.9938	0.02/0.40	0.004/0.08	6.39	11.81	8.53
ANC	0.02–50	0.9925	0.02/0.40	0.004/0.08	3.44	4.58	5.63
PYR	0.05–50	0.9921	0.05/1.00	0.014/0.28	6.24	7.06	8.82

^a^ ng mL^−1^ was achieved with spiked solution and ng g^−1^ was corresponding concentration for soil.

**Table 2:** The molecular formula, structure, logK_ow_ and *EF*s that obtained with the developed method.

Analyte	Molecular Formula	Structure	logK_ow_	*EF*s
ACE	C_12_H_10_	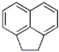	3.92	3000
FLU	C_13_H_10_	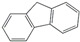	4.18	3278
PHE	C_14_H_10_	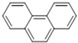	4.46	4327
ANC	C_14_H_10_	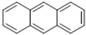	4.45	4398
PYR	C_16_H_10_	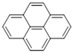	4.88	1526

**Table 3:** The contents of target PAHs in real samples and recoveries.

Analyte	Campus Soil			Zhengkai Avenue Soil		
	Found (ng g^−1^)	Added (ng g^−1^)	Recovery, % (RSD, %)	Found (ng g^−1^)	Added (ng g^−1^)	Recovery, % (RSD, %)
ACE	N.D.	10	120.0 (2.6)	N.D.	10	116.0 (5.9)
		100	86.0 (2.5)		100	80.0 (3.0)
		400	81.2 (0.4)		400	92.0 (2.0)
FLU	16.2	10	80.0 (5.5)	15.2	10	80.0 (4.0)
		100	80.0 (3.4)		100	89.8 (5.6)
		400	113.4 (3.1)		400	97.6 (9.8)
PHE	12.0	10	83.7 (4.7)	12.2	10	80.0 (2.5)
		100	80.0 (3.5)		100	80.2 (1.7)
		400	92.0 (6.5)		400	89.4 (7.9)
ANC	6.4	10	81.4 (2.4)	5.6	10	82.8 (4.0)
		100	103.0 (5.1)		100	80.0 (0.6)
		400	84.9 (1.9)		400	78.0 (4.0)
PYR	255.6	10	80.1 (5.8)	251.0	10	79.9 (0.5)
		100	86.8 (5.8)		100	94.3 (1.9)
		400	89.0 (0.1)		400	82.7 (2.8)

N.D.: Not detected.

**Table 4:** Comparison of the proposed method with other reported techniques for determination of PAHs in soil.

Sorbents	Extraction Methods	Detection Techniques	Linearity Range (ng g^−1^)	LODs (ng g^−1^)	Thermal Stability (°C)	Refs.
Fe_3_O_4_@mSiO_2_-Ph-PTSA ^a^	MSPE	GC-MS	5–500	0.07–0.41	N.P. ^b^	[56]
PDMS	SPME	GC-MS	40–4000	4.2–8.5	N.P.	[57]
Carbon nanospheres	SPME	GC-FID	6.0–2700	1.53–2.70	350	[58]
BN@rGO	SPME	GC-FID	1.0–400	0.3–0.5	400	[59]
MWCNTs/MnO_2_/PEDOT ^c^	SPME	GC-FID	0.5–250	0.1–0.8	300	[60]
h-BN	SPME	GC-FID	0.4–1000	0.08–0.66	800	This work

^a^ phenyl-modified magnetic mesoporous silica, ^b^ not provided, ^c^ multi-walled carbon nanotubes/manganese dioxide nanocomposite-based polythiophene.

## Data Availability

Not applicable.
